# Resection of swollen temporal muscles in patients with intractable intracranial hypertension after decompressive craniectomy

**DOI:** 10.1007/s00701-021-04718-1

**Published:** 2021-01-25

**Authors:** Shih-Hao Huang, Abel Po-Hao Huang, Sheng-Jean Huang, Lu-Ting Kuo

**Affiliations:** 1 Division of Neurosurgery, Department of Surgery, Far Eastern Memorial Hospital, New Taipei City, Taiwan; 2 Department of Healthcare Administration, Oriental Institute of Technology, New Taipei City, Taiwan; 3 Division of Neurosurgery, Department of Surgery, National Taiwan University Hospital, No. 1, Changde St., Zhongzheng Dist, Taipei City, 10048 Taiwan

**Keywords:** Clinical outcome, Craniectomy, Head injury, Temporal muscle

## Abstract

**Background:**

Decompressive craniectomy is employed as treatment for traumatic brain swelling in selected patients. We discussed the effect of temporal muscle resection in patients with intractable intracranial hypertension and temporal muscle swelling after craniectomy.

**Methods:**

Records of 280 craniectomies performed on 258 patients who were admitted with severe head injury were retrospectively reviewed. Eight patients developed intractable increased intracranial pressure with temporal muscle swelling within 24 h after craniectomy and were treated by muscle resection.

**Results:**

The initial Glasgow Coma Scale score was 7 ± 1. The mean intracranial pressure was 41.7 ± 8.59 mmHg before muscle resection and 14.81 ± 8.07 mmHg immediately after surgery. Five patients had skull fracture and epidural hematoma at the craniectomy site. The mean intensive care unit stay was 11.25 ± 5.99 days. Glasgow Outcome Scale-Extended scoring performed during the 12-month follow-up visit showed that 6 patients (75%) had a favorable outcome.

**Conclusions:**

Our study findings indicate that a direct impact on the temporal region during trauma may lead to subsequent temporal muscle swelling. Under certain circumstances, muscle resection can effectively control intracranial pressure.

## Introduction

Intracranial hypertension secondary to cerebral edema is a major problem in patients with traumatic brain injury (TBI). The control of increased intracranial pressure (ICP) for the prevention of secondary brain injury is a key goal in the treatment of TBI [2, 11]. Decompressive craniectomy has been reported to be effective in the management of traumatic brain swelling when conservative treatment fails [16]. Bleeding and infection are well recognized to be general complications of decompressive craniectomy during the acute stage. Nevertheless, we observed temporal muscle swelling as an uncommon complication of decompressive craniectomy. In this study, we retrospectively evaluated the outcomes of temporal muscle resection as treatment for postcraniectomy intracranial hypertension due to marked temporal muscle swelling.

## Methods

### Inclusion criteria

The study sample comprised patients who underwent decompressive craniectomy and expanded duraplasty after TBI at the National Taiwan University Hospital and its Yunlin branch. This study was approved by the Committee on Human Studies at the National Taiwan University Hospital, and written consent for the publication of findings was obtained from patients or their families. All procedures performed were in accordance with the ethical standards of the institutional research committee and with the 1964 Helsinki Declaration and its later amendments or comparable ethical standards.

Prehospital management was completed according to the standards of the Taiwan Society of Emergency Medicine, which are compatible with the Brain Trauma Foundation’s Guidelines for Prehospital Management of Traumatic Brain Injury. On hospital arrival, patients were examined and further treated in accordance with the advanced trauma life support guidelines and the American Association of Neurological Surgeons/Congress of Neurological Surgeons Guidelines for the Management of Severe Head Injury. The initial functional status of patients was evaluated by emergency physicians using the Glasgow Coma Scale (GCS). All patients underwent cerebral computed tomography (CT) scan after injury. The decision to operate on an acute subdural hematoma, epidural hematoma, or other intracranial hemorrhages was based on patients’ GCS score, pupillary exam, comorbidities, CT findings, age, and the presence of neurological deterioration over time. Decompressive surgery was performed with intraparenchymal placement of ICP catheters if patients presented with acute neurological deterioration immediately after the accident or intractable intracranial hypertension after medical treatment and if the CT scan revealed diffuse intracerebral lesions or brain edema with brain stem compression. Standard decompressive craniectomy procedure included removal of a large fronto-temporo-parietal bone flap measuring more than 12 × 13 cm, and duraplasty was carried out following wide durotomy with a temporal fascia flap or an artificial dural substitute. One or two subgaleal closed wound vacuum (CWV) drains were routinely placed. All patients were admitted to the intensive care unit (ICU) after surgery.

### Data collection

Standard monitoring of these ICU patients included invasive measurement of arterial blood pressure (zero point in the fourth intercostal space at the midclavicular line), pulse oximeter oxygen saturation, end-tidal CO_2_ concentration, and tympanic body temperature. All patients were treated with 30-degree head elevation and monitored by neurological assessment, including hourly assessment of pupillary reflex to light. Intraparenchymal ICP monitoring was instituted in all cases by an ICP microsensor (Codman Electrode MicroSensor; Johnson & Johnson Medical Ltd., New Brunswick, NJ, USA) or a fiberoptic ICP monitor (model 110-4BT; Camino Laboratories, San Diego, CA, USA). ICP was measured continuously and recorded hourly. The therapy is aimed at maintaining ICP at less than 20 mmHg and cerebral perfusion pressure at more than 60 mmHg. Mannitol, the most commonly used hyperosmolar agent for treating intracranial hypertension, was used, and its dosage was adjusted according to the ICP. In addition, 3% hypertonic saline was given to help reduce ICP. Some patients were sedated to prevent an increase in ICP caused by agitation, posturing, or coughing. Postoperative CT scan of the head was conducted when the ICP was persistently higher than 20 mmHg or at 3 days after craniectomy. Surgical resection of the temporal muscle was performed if the ICP was still above 20 mmHg despite medical treatment and if on postoperative CT the swollen muscle demonstrated significant mass effect, as judged by the attending neurosurgeons.

### Outcome measurements

A neurosurgeon evaluated the outcomes in the outpatient clinic using the Glasgow Outcome Scale-Extended (GOSE) score at 12 months in accordance with published guidelines [21]. The GOSE is an 8-point scale in which 1 indicates death; 2, vegetative state; 3, lower severe disability; 4, upper severe disability; 5, lower moderate disability; 6, upper moderate disability; 7, lower good recovery; and 8, upper good recovery. The structured interview for determining the GOSE score was developed by Wilson et al. in 1998 and validated by Pettigrew et al. in 2003 to improve the reliability of the evaluation [15, 21]. Other variables considered in this study included age, sex, mechanism of injury, GCS at admission, vital signs, type of pathology, ICP, TCCS findings, and postoperative complications.

### Statistical analysis

Data were entered into a computerized database and analyzed using SPSS for Windows version 14.0 (SPSS Inc., Chicago, IL, USA). ICP and TCCS findings are presented as mean ± standard deviation. The ICPs before and after the second operation with temporal muscle excision were compared using a paired *t*-test. Statistical differences were considered significant at *p* < 0.05.

## Results

**Fig. 1:**
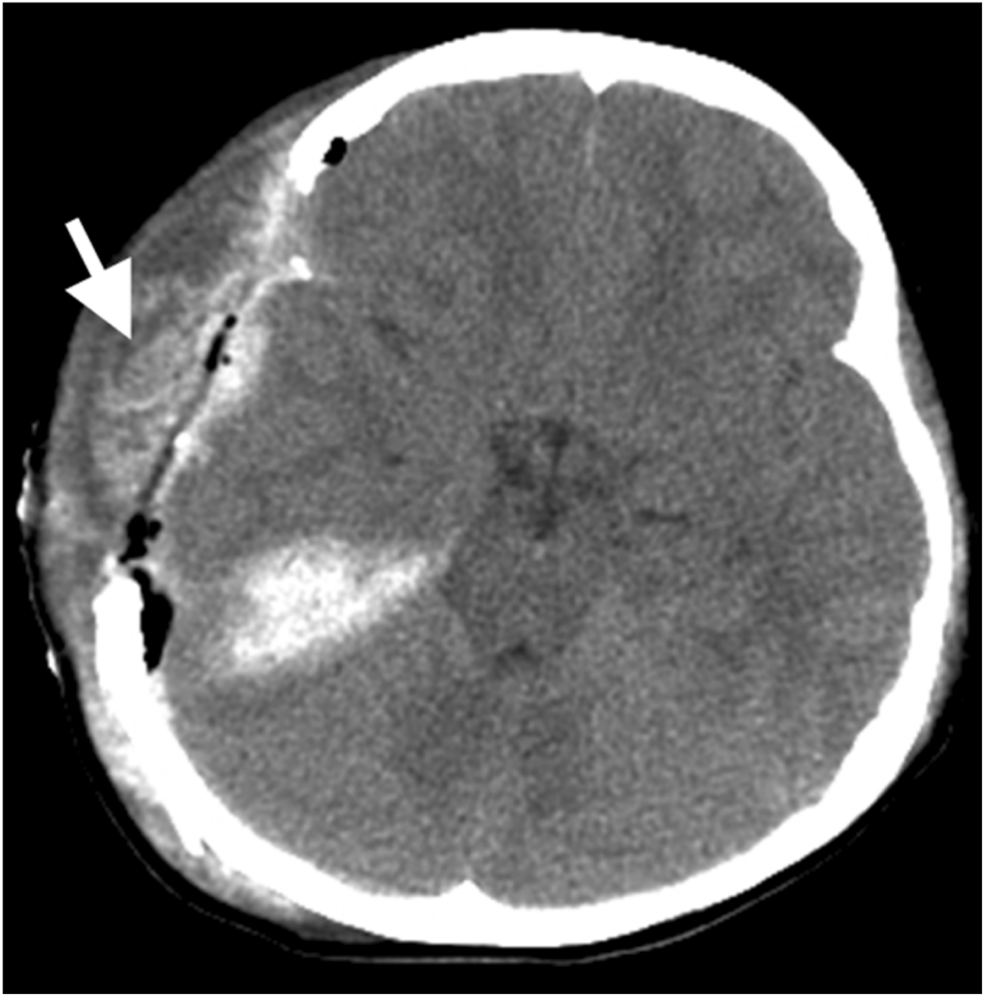
Swollen and thickening temporal muscle (white arrow) caused a mass effect and compressed the basal cistern

**Fig. 2:**
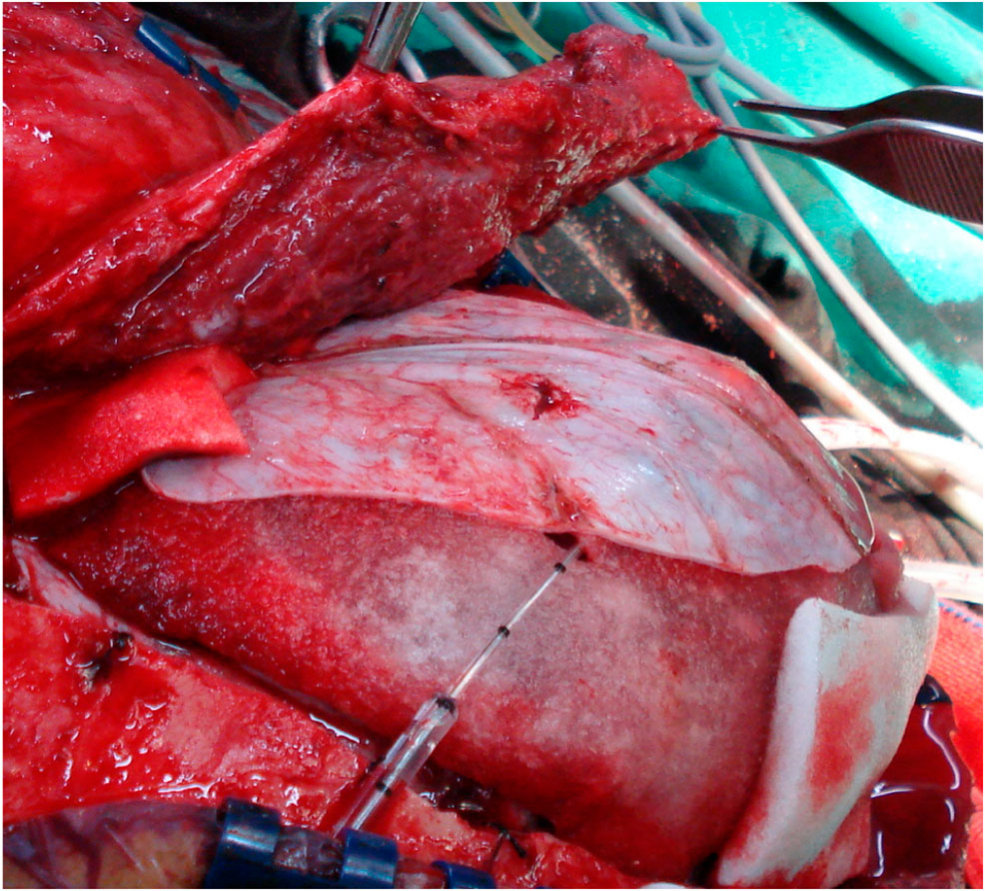
Intraoperative photograph showing the swollen temporal muscle and fascia elevated from the temporal fossa

**Fig. 3:**
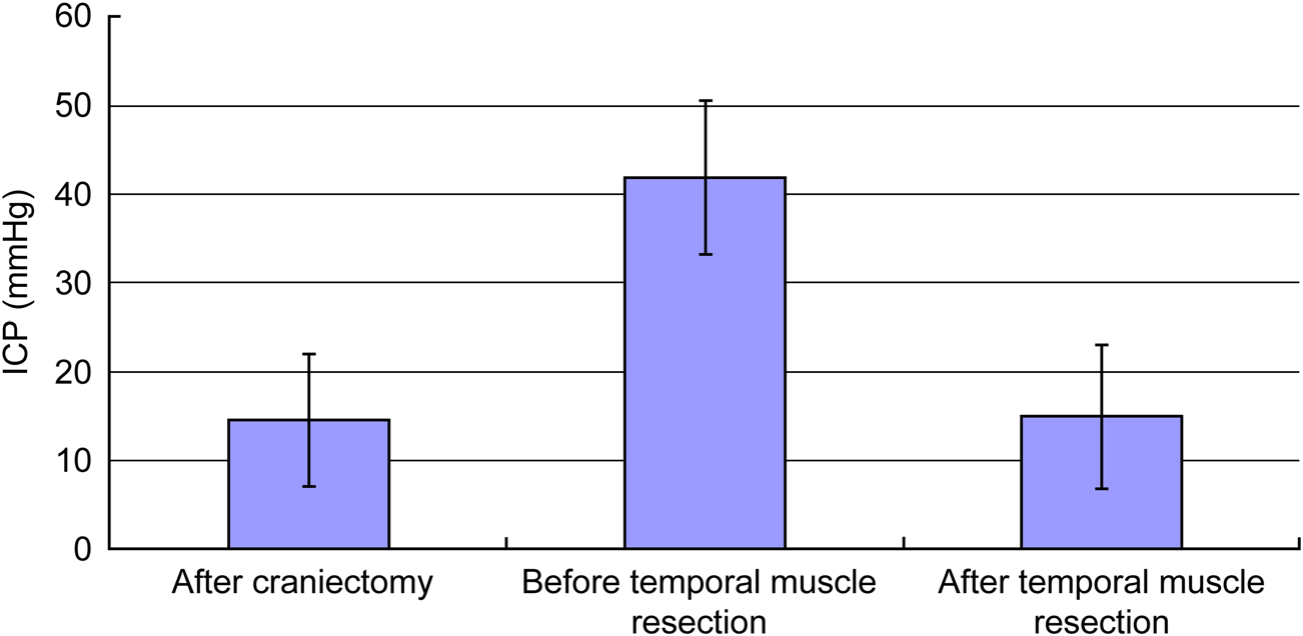
ICP changes after craniectomy, before temporal muscle resection, and after temporal muscle resection. *ICP* intracranial pressure

A total of 280 craniectomies were performed on 258 patients (174 men, 84 women; mean age: 43.5 ± 19.6 years) with moderate-to-severe TBI. Assessment of 1-year functional outcome indicated favorable outcome (GOSE 6–8) for 40.3% of patients. Eight patients (6 men, 2 women; 2.9% of all craniectomies) developed intractable intracranial hypertension and brain stem compression within 24 h after decompressive craniectomy due to temporal muscle swelling (Fig. 1). One or two subgaleal CWV drains were routinely placed according to the intraoperative findings of oozing from the muscle and fascia, and no statistical difference in the number of drains placed was noted between these eight patients and the general cohort. There was little new postoperative epidural or subdural blood clot formation. Consequently, the temporal muscle and its fascia at the level of the middle cranial fossa were excised without enlargement of the craniectomy window (Fig. 2). The thickness of the transected muscle was 2.35 ± 0.33 cm. The patency of one or two CWV drains placed during the first surgery was confirmed. Brain injury in all of these cases was due to traffic accidents. The age of these eight patients was 50.57 ± 12.19 years, whereas their initial GCS score was 7 ± 1. Traumatic subdural hematoma, contusional intracerebral hemorrhage, and skull bone fracture were detected on CT scans in six, seven, and five patients, respectively (Table 1). Temporal muscle excision relieved brain stem compression. The mean ICP recorded within 1 h after temporal muscle excision was significantly lower than the ICP recorded immediately before the second surgery (14.81 ± 8.07 mmHg vs. 41.70 ± 8.59 mmHg, *p* < 0.05) (Fig. 3). The ICP after temporal muscle resection ranged from 6 to 27 mmHg, and 6 patients had their ICP reduced to below 20 mmHg after the second surgery. ICP was monitored and recorded hourly for ≥7 days after the initial surgery, and the mean ICU stay was 11.25 ± 5.99 days.

**Table 1:** Demographic characteristics and functional outcomes of patients

Case no.	Age	Sex	GCS before craniectomy	Diagnosis	TM thickness (cm)	GOSE (12 months)
1	48	M	E2M5V2	Contusion	3	8
2	55	M	E1M4V2	Skull fx, EDH, SDH, SAH	2.3	1
3	58	F	E3M5V2	Skull fx, EDH, SDH, contusion	2.5	4
4	34	M	E3M5V3	Skull fx, EDH, SDH, contusion	3	6
5	36	M	E1M4V1	SDH, contusion	2	7
6	68	M	E1M4V1	Contusion	3	8
7	55	M	E1M4V2	Skull fx, EDH, SDH, SAH, contusion	2.5	5
8	19	F	E2M5V3	Skull fx, EDH, SDH, contusion	1.9	8

*EDH* epidural hematoma, *fx* fracture, *GCS* Glasgow Coma Scale; *GOSE (12 months)* Glasgow Outcome Scale-Extended at 12 months after trauma, *SAH* subarachnoid hemorrhage, *SDH* subdural hematoma, *TM* temporal muscle

Cranioplasty was performed, on average, 4.9 months later. One patient developed hydrocephalus and was treated with ventriculoperitoneal shunt placement. Muscle resection after cranioplasty resulted, to some extent, in an asymmetric appearance of the face in all patients. None of the patients reported any limitation of mouth opening or masticatory dysfunction. Only one patient had intermittent soreness and pain at the temporomandibular joint.

One patient died in the ICU due to respiratory failure. As shown in Table 1, the long-term outcomes (GOSE scores obtained at follow-up in the outpatient clinic at 1 year after admission) demonstrated that all patients improved over time, with three patients having a GOSE score of 8 and two patients having scores lower than 5.

## Discussion

This study presented the details of eight patients with intractable intracranial hypertension due to temporal muscle swelling after decompressive craniectomy in a cohort of 258 patients on whom 280 craniectomies were performed. Temporal muscle excision was performed to yield good ICP control. One-year follow-up demonstrated favorable outcome in six patients. To the best of our knowledge, this is the first study to evaluate the long-term outcome of patients treated using temporal muscle resection after craniectomy. Our findings suggest an option for treating this critical condition and achieving a favorable outcome.

No controlled clinical trial has been conducted to prove that decompressive craniectomy is more effective than maximal medical therapy in improving functional outcomes. Nevertheless, many studies have indicated the efficacy of decompressive craniectomy in directly reducing the ICP [4, 6, 12, 16, 19]. Several recent studies have shown that decompressive craniectomy can considerably decrease the ICP within 24 h, thereby improving cerebral perfusion, preventing ischemic damage, and avoiding brain herniation [12, 16, 19, 24]. In other conditions that cause brain edema such as malignant cerebral infarction, tumors, and subarachnoid hemorrhage, the temporary removal of a piece of skull bone reduced the mortality rate and improved the outcome in some cases [4, 5, 8, 17]. The size of the craniectomy should be at least 12 cm [9, 27]. The current guidelines recommend a bone flap measuring at least 12 × 15 cm [3]. If the head size is taken into account, a bone flap circumference over skull hemi-circumference of >65% is usually sufficient [18].

Complications can occur due to not only decompressive craniectomy in the acute stage but also delayed cranioplasty. For instance, a review of 300 cases of cranioplasty for various intracranial pathologies mentioned several minor subsequent problems, including skull deformity, subgaleal effusion, infection requiring bone flap removal, and repeated cranioplasty [23]. Additionally, Yoo et al. reported incidence rates of 13% for subdural hygroma requiring burr hole drainage and 13% for postcranioplasty infection [24]. In another study, craniectomy performed on 60 patients was complicated by ischemic and hemorrhagic areas that formed next to the edge of the bony defect, as observed on postoperative CT scans [20]. After craniectomy, epidural hematoma can render decompression ineffective; this may be caused by oozing from the wound or bone edge and may be exacerbated by temporal muscle swelling. Although there exists one report of suboptimal decompression after craniectomy in a patient with middle cerebral artery infarction due to temporal muscle swelling and intramural hematoma [1], no other studies have described this complication as a potential cause of ICP elevation after craniectomy. A study involving 15 patients with malignant cerebral infarction suggested that compared to conventional craniectomy, temporal muscle resection together with decompressive craniectomy improved the outcome, minimally affected masticatory function, and doubled the amount of extracranial space for the brain [14].

Our study included eight patients who exhibited ICP elevation due to swollen temporal muscles within 24 h after craniectomy. These eight patients accounted for only 2.9% of the 258 craniectomies performed during the study period. Thus, it seems that sufficient decompression can be achieved without temporal muscle resection in most cases. CT scans confirmed the diagnosis of a swollen temporal muscle with/without intramural hematoma for these eight patients. ICP considerably decreased after muscle resection. Five (62.5%) of these patients had skull fracture and epidural hematoma at the craniectomy site, and subcutaneous hematoma and swollen temporal muscle were diagnosed during craniectomy. The remaining three patients (37.5%) had only subdural hematoma and brain contusion. Therefore, we suggest that direct soft tissue injury during head trauma and iatrogenic manipulation might be possible risk factors for progressive temporal muscle swelling after craniectomy. Coagulopathy, which is another possible risk factor, has been previously proposed [1]; however, the coagulation profiles were all normal in our eight patients. To prevent uncontrolled ICP elevation after surgery, we suggest considering temporal muscle resection if craniectomy is performed on the site of direct impact during the head injury and the temporal muscle is severely swollen during surgery.

Anatomically, the temporal muscle (a masticatory muscle) arises from the temporal fossa and is covered by the temporal fascia. It passes medial to the zygomatic arch, attaches to the coronoid process of the mandible, and acts to elevate the mandible and close the jaw. The temporal muscle is innervated by deep temporal nerves from the mandibular branch of the trigeminal nerve, and the blood supply to this muscle comes from deep temporal arteries, which pass between the temporal muscle and pericranium. Temporal muscle swelling after craniectomy may result in additional mass effect and brain compression. To avoid such complication, extensive resection of the temporal muscle and fascia down to the zygomatic arch has been proposed [14, 25]. Zhang et al. found that temporal muscle resection provides an additional decompression volume of 26.5 cm^3^ [26]. However, the temporal muscle is the most important muscle of the temporomandibular joints involved in mastication, and temporal muscle resection could result in functional and cosmetic problems. Surprisingly, the mastication function may only be minimally affected. In a study of patients with cerebral infarction who underwent craniectomy and temporal muscle resection, there were no differences in pain and mouth opening [14]. Although the maximal bite force was decreased, the chewing function was intact. Measurement of the bite force produced by jaw elevators is a way to evaluate the functioning of the masticatory system [7]. Current bite force recording devices include the gnathodynamometer, deformation-sensitive piezoelectric film, and electromyography [10, 13]. It has been shown that the masseter and medial pterygoid muscle could compensate for the decreased bite force because the grinding phase of the closure stroke requires only one-third of the maximal bite force [6]. Taking into account cosmetic issues, transplantation of a free autologous fat graft is a well-accepted procedure to restore contour in the soft tissue. To fill the soft tissue defect resulting from temporal muscle resection, fat harvested from the lower abdomen or thigh should be considered for use in grafting after cranioplasty [22]. Alternatively, hyaluronic acid could be administered via injection. However, repeated treatments are indicated due to the unpredictable time course of resorption after both of these cosmetic procedures.

This study has some limitations. First, the heterogeneity of the patients’ characteristics including age, comorbidities, and the type of head injury (the location and volume of intracranial hemorrhage, etc.) may affect the postoperative outcomes. Second, the study included a relatively small number of patients with temporal muscle swelling, making statistical analysis unfeasible. Despite the abovementioned limitations, understanding the choice of muscle resection in selected cases has clinical implications in the management of intractable ICP due to temporal muscle swelling.

## Conclusions

Our study findings indicate that a direct impact on the temporal region during trauma may lead to subsequent temporal muscle swelling. Under certain circumstances, a second surgery with temporal muscle resection could effectively control intractable ICP after craniectomy.

## Data Availability

The datasets used and/or analyzed during the current study are available from the corresponding author on reasonable request.

## Abbreviations

CT: Computed tomography
CWV: Closed wound vacuum
GCS: Glasgow Coma Scale
GOSE: Glasgow Outcome Scale-Extended
ICP: Intracranial pressure
ICU: Intensive care unit
TBI: Traumatic brain injury
TCCS: Transcranial color-coded sonography
